# Clinical Outcomes of Selenium Supplementation in Hashimoto's Thyroiditis Without Selenium Deficiency: A Large‐Scale Retrospective Cohort Study

**DOI:** 10.1002/edm2.70239

**Published:** 2026-05-18

**Authors:** Eman A. Toraih, Sarah Yaghi, Claire Ardis, Lori Tran, Ahmed Abdelmaksoud, Rami M. Elshazli, Mohammad H. Hussein, Hinali Patel, Ekramy M. Elmorsy, Jessan A. Jishu, Hani Aiash, Manal S. Fawzy

**Affiliations:** ^1^ Genetics Unit, Department of Histology and Cell Biology Suez Canal University Faculty of Medicine Ismailia Egypt; ^2^ SUNY Upstate Medical University Syracuse New York USA; ^3^ Tulane University School of Medicine New Orleans Louisiana USA; ^4^ Department of Internal Medicine University of California Riverside California USA; ^5^ Department of Basic Sciences, Faculty of Physical Therapy Horus University—Egypt New Damietta Egypt; ^6^ Department of Biological Sciences, Faculty of Science New Mansoura University New Mansoura City Egypt; ^7^ Ochsner Clinic Foundation New Orleans Louisiana USA; ^8^ Louisiana State University Health Sciences Center New Orleans Louisiana USA; ^9^ Center for Health Research Northern Border University Arar Saudi Arabia

**Keywords:** autoimmune, Hashimoto's thyroiditis, propensity score matching, selenium, TriNetX

## Abstract

**Introduction:**

Selenium supplementation has been suggested as a therapeutic option for Hashimoto's thyroiditis, but its effects on thyroid function and other patient‐related outcomes remain unclear.

**Methods:**

This retrospective cohort study utilised a global federated health research network to compare patients with Hashimoto's thyroiditis who received selenium supplementation after diagnosis to those who did not. Propensity score matching was performed to balance baseline characteristics. Primary outcomes included longitudinal thyroid function measures, while secondary outcomes included development of autoimmune diseases, thyroid cancer, need for thyroidectomy and all‐cause mortality. All outcomes were followed for 5 years.

**Results:**

After matching, 2347 patients were included in each cohort. Selenium supplementation was associated with persistently higher mean TSH, free T4 and TPOAb levels, as well as greater proportions of elevated TPOAb (> 35 IU/mL) at 3 years (6.9% vs. 5.0%, *p* = 0.01) and 5 years (7.9% vs. 5.3%, *p* < 0.01). The incidence of autoimmune disorders was higher in the selenium group (RR 1.33, 95% CI 1.14–1.56), particularly Sjögren's syndrome (RR 1.61, 95% CI 1.03–2.52) and psoriasis (RR 2.69, 95% CI 1.52–4.76). The rates of thyroid cancer and need for thyroidectomy were similar between groups. However, selenium use was associated with increased all‐cause mortality (RR 1.38, 95% CI 1.09–2.03).

**Conclusion:**

Selenium supplementation in Hashimoto's thyroiditis was associated with worsened biochemical autoimmunity, increased autoimmune comorbidity and higher mortality. These findings should be interpreted cautiously given the absence of baseline selenium measurements, which limits assessment of deficiency status. Nevertheless, they highlight the need for further research to confirm these results.

## Graphical abstract

Selenium supplementation among patients with Hashimoto's thyroiditis does not benefit thyroid biomarkers and can worsen autoimmune comorbidities, especially when they are not selenium‐deficient to begin with.

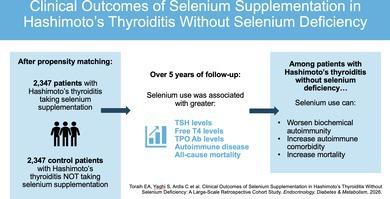

## 1 Introduction

Hashimoto's thyroiditis (HT) is a chronic autoimmune disorder characterised by the gradual destruction of the thyroid gland, leading to hypothyroidism. It is the most common cause of primary hypothyroidism in iodine‐sufficient areas, affecting approximately 5% of the general population, with a higher prevalence in women [1, 2]. While the standard treatment for HT is thyroid hormone replacement therapy, which aims to correct the hormonal imbalance, recent studies have suggested that selenium supplementation may play a beneficial role in managing the disease [3, 4, 5, 6].

Selenium is an essential trace mineral that is crucial in thyroid hormone synthesis and metabolism [7]. It is incorporated into selenoproteins, such as glutathione peroxidase and thioredoxin reductase, which protect the thyroid gland from oxidative stress [8]. Selenium deficiency has been associated with an increased risk of autoimmune thyroid disorders, including HT [9]. Indeed, studies have suggested that selenium supplementation may help reduce thyroid antibodies, improve thyroid function and alleviate symptoms in affected patients [10, 11, 12, 13].

While selenium supplementation has shown promise in the management of HT, the impact among patients with no documented selenium deficiency or those who use it without clear medical indication is understudied. In these patients, there may be a high risk of developing other autoimmune disorders, as evidence has been shown for rheumatoid arthritis, systemic lupus erythematosus, or celiac disease [14]. Moreover, selenium supplementation, particularly through the activity of selenoproteins, may play a role in the prevention and treatment of thyroid cancer and may potentially reduce the incidence of other cancers in patients with HT; however, outcomes related to cancer development in patients without documented selenium deficiency remain poorly characterised [15, 16].

This study aims to investigate the effects of selenium supplementation on the course of HT, its prognosis, and the risk of developing comorbidities, particularly other autoimmune diseases and thyroid cancer, among patients without documented selenium deficiency. These findings may help clarify the potential role of selenium supplementation in the management of HT and inform clinical decision‐making regarding its use.

## 2 Methods

### 2.1 Data Source and Study Population

This retrospective cohort study utilised the TriNetX database, a global federated health research network, to compare patients with HT who received selenium supplementation after diagnosis to those who did not. TriNetX aggregates clinical information primarily from insured individuals receiving healthcare across a variety of institutions worldwide, including community hospitals and tertiary centers [17]. Diagnostic codes from the International Classification of Diseases, 10th Revision (ICD‐10) were obtained directly from participating healthcare organizations (HCOs). The study population included patients aged 18 years or older with a confirmed diagnosis of HT and at least one of the following laboratory criteria: (a) elevated thyroid peroxidase antibodies (TPOAb) > 35 IU/mL or (b) elevated thyroglobulin antibodies (TgAb) > 115 IU/mL. Patients were divided into two cohorts: those who received selenium supplementation within 6 months of diagnosis and those who did not (Table S1).

### 2.2 Propensity Scores Matching

Propensity score matching was employed to balance the two cohorts based on age, sex, race, ethnicity, body mass index (BMI), nicotine dependence, alcohol‐related disorders, prior thyroidectomy and the presence of other autoimmune comorbidities at baseline. Covariates for propensity score matching were selected based on their potential influence on the outcomes of interest and to ensure equal probability of receiving selenium supplementation among groups with the same level of bias‐adjusted propensity scores.

### 2.3 Primary Outcomes

The primary outcomes of interest involved the prognosis of HT as measured by changes in thyroid function tests at 6 months, 1 year, 3 years and 5 years after the initiation of selenium supplementation or the corresponding time points for the non‐supplementation cohort. The following thyroid function tests were evaluated: (a) thyroid‐stimulating hormone (TSH) levels, (b) free thyroxine (T4) levels, (c) TPOAb levels and (d) TgAb levels.

### 2.4 Secondary Outcomes

Secondary outcomes included the risk of developing new autoimmune diseases and the onset of these diseases, as identified by ICD‐10 coding. The autoimmune diseases evaluated include rheumatoid arthritis, systemic lupus erythematosus, multiple sclerosis, type 1 diabetes mellitus, celiac disease, pernicious anaemia, Addison's disease, Sjögren's syndrome, vitiligo and psoriasis. Additionally, the risk of developing thyroid cancer and all‐cause mortality was evaluated after the initiation of selenium supplementation or the corresponding time points for the non‐supplementation cohort (Table S2). Similarly, all outcomes were evaluated at 6 months, 1 year, 3 years and 5 years.

### 2.5 Statistical Analysis

Descriptive statistics were presented as means ± standard deviations for continuous variables and frequencies (percentages) for categorical variables. Two‐sided Chi‐square tests were used to compare categorical characteristics between the two cohorts. In a large cohort size, the sampling distribution of the mean can be approximated as normal according to the central limit theorem, supporting the use of parametric comparisons for continuous data. Logistic regression was employed to assess the risk of developing comorbidities, adjusting for potential confounders such as age, sex and baseline autoimmune comorbidities. Relative risks (RRs) and 95% confidence intervals were reported. Survival analysis techniques, such as Kaplan–Meier curves and Cox proportional hazards models, were used to compare the time to the onset of autoimmune diseases between the two cohorts. Statistical analyses were conducted using the TriNetX built‐in tools, and a *p*‐value of less than 0.05 was considered statistically significant.

## 3 Results

### 3.1 Study Population

From a global collaborative network of 120 HCOs across 17 countries, 146,940,654 patients were identified, of which 125,149,668 were adults. Among the adult patients, 488,416 individuals with HT were identified. Patients with dietary selenium deficiency (*n* = 311), prior excision of the thyroid gland (*n* = 12,271) and neoplasm (*n* = 167,288) at the time of HT diagnosis were excluded. The final study population consisted of 308,546 individuals, with 2464 adults ≥ 18 years old in the treated group (selenium supplementation after HT diagnosis) from 63 HCOs and 306,082 adults ≥ 18 years old in the control group (no selenium supplementation) from 117 HCOs.

Before matching, the treated group (*n* = 2464) and the control group (*n* = 306,082) differed significantly in several demographic variables, including age, sex, race and ethnicity (all *p* < 0.01). The treated group also had a significantly higher proportion of individuals with a personal history of irradiation (*p* < 0.01), type 1 diabetes mellitus (*p* = 0.01) and pernicious anaemia (*p* = 0.01) compared to the control group.

After propensity score matching, the treated and control groups comprised 2347 individuals. The matching process successfully balanced the two groups across all 27 characteristics, with no significant differences observed between the groups (all *p* > 0.05) (Table 1).

**TABLE 1:** Baseline characteristics of the study population.

Characteristic	Before propensity matching			After propensity matching		
	Treated (*n* = 2464)	Not treated (*n* = 306,082)	*p*	Treated (*n* = 2347)	Not treated (*n* = 2347)	*p*
Demographics						
Age at index	45.0 ± 18.4	42.7 ± 18.9	**< 0.01**	45.0 ± 18.4	44.8 ± 18.3	0.79
Female	2038 (86.8%)	240,956 (81.3%)	**< 0.01**	2038 (86.8%)	2039 (86.9%)	0.97
Male	269 (11.5%)	41,018 (13.8%)	**< 0.01**	269 (11.5%)	271 (11.5%)	0.93
White	1341 (57.1%)	197,467 (66.7%)	**< 0.01**	1341 (57.1%)	1345 (57.3%)	0.91
Black/African American	91 (3.9%)	10,962 (3.7%)	0.65	91 (3.9%)	78 (3.3%)	0.31
Asian	35 (1.5%)	10,821 (3.7%)	**< 0.01**	35 (1.5%)	34 (1.4%)	0.90
Other Race	49 (2.1%)	12,676 (4.3%)	**< 0.01**	49 (2.1%)	47 (2%)	0.84
Not Hispanic or Latino	1273 (54.2%)	186,586 (63%)	**< 0.01**	1273 (54.2%)	1256 (53.5%)	0.62
Hispanic or Latino	95 (4.0%)	21,052 (7.1%)	**< 0.01**	95 (4.0%)	105 (4.5%)	0.47
Nicotine dependence	85 (3.6%)	9837 (3.3%)	0.42	85 (3.6%)	78 (3.3%)	0.58
Alcohol related disorders	19 (0.8%)	2178 (0.7%)	0.68	19 (0.8%)	18 (0.8%)	0.87
Body mass index, kg/m^2^	28.1 ± 12.1	30.0 ± 190.6	0.78	28.1 ± 12.1	29.2 ± 12.4	0.07
Personal history of irradiation	10 (0.4%)	199 (0.1%)	**< 0.01**	10 (0.4%)	10 (0.4%)	1.00
Socioeconomic problems	32 (1.4%)	3657 (1.2%)	0.57	32 (1.4%)	27 (1.2%)	0.51
Free thyroxine (T4), ug/dL	8.1 ± 2.8	8.1 ± 2.8	0.94	8.1 ± 2.8	8.1 ± 2.7	0.94
Autoimmune comorbidities						
Rheumatoid arthritis	10 (0.4%)	952 (0.3%)	0.37	10 (0.4%)	10 (0.4%)	1.00
Other rheumatoid arthritis	37 (1.6%)	4085 (1.4%)	0.41	37 (1.6%)	36 (1.5%)	0.91
SLE	17 (0.7%)	2525 (0.9%)	0.50	17 (0.7%)	16 (0.7%)	0.86
Type 1 diabetes mellitus	64 (2.7%)	10,975 (3.7%)	**0.01**	64 (2.7%)	57 (2.4%)	0.52
Multiple sclerosis	10 (0.4%)	1295 (0.4%)	0.94	10 (0.4%)	10 (0.4%)	1.00
Celiac disease	21 (0.9%)	2300 (0.8%)	0.52	21 (0.9%)	22 (0.9%)	0.88
Pernicious anaemia	11 (0.5%)	667 (0.2%)	**0.01**	11 (0.5%)	10 (0.4%)	0.83
Addison's disease	10 (0.4%)	936 (0.3%)	0.35	10 (0.4%)	10 (0.4%)	1.00
Sjögren syndrome	20 (0.9%)	2700 (0.9%)	0.76	20 (0.9%)	17 (0.7%)	0.62
Vitiligo	10 (0.4%)	945 (0.3%)	0.36	10 (0.4%)	10 (0.4%)	1.00
Psoriasis	19 (0.8%)	2523 (0.9%)	0.82	19 (0.8%)	13 (0.6%)	0.29
Family history of malignant cancer	55 (2.3%)	7459 (2.5%)	0.59	55 (2.3%)	56 (2.4%)	0.92

*Note:* Data are presented as number (percentage) or mean ± standard deviation. Bold values indicate statistical significance set at *p* < 0.05. Statistical significance set at *p* < 0.05.

Abbreviation: SLE, systemic lupus erythematosus.

### 3.2 Effects of Selenium Supplementation on Thyroid Function and Autoantibodies

Selenium supplementation in patients with HT was associated with a higher proportion of patients with elevated TPOAb and consistently higher mean T4, TPOAb and TSH levels than the control group over the 5‐year study period.

The proportion of patients with elevated thyroid peroxidase antibodies (TPOAb > 35 IU/mL) was consistently higher in the selenium‐treated group compared to the control group at all time points (Table 2). This difference reached statistical significance at 3 years (6.9% vs. 5.0%, *p* < 0.01) and remained significant at 5 years (7.9% vs. 5.3%, *p* < 0.01). In contrast, the proportion of patients with elevated thyroglobulin antibodies (TgAb > 115 IU/mL) was similar between the two groups throughout the study, with no significant differences observed at any time point. Regarding elevated thyroid‐stimulating hormone (TSH > 4.5 mIU/L), the treated group demonstrated a lower proportion of patients with elevated levels at 3 years (7.3% vs. 9.2%, *p* = 0.02), which persisted at 5 years (8.5% vs. 10.2%, *p* = 0.05).

**TABLE 2:** Trends in the proportion of patients with elevated thyroid markers.

Timeline	High TPOAb (> 35 IU/mL)		*p*	High TgAb (> 115 IU/mL)		*p*	High TSH (> 4.5 mIU/L)		*p*
	Treated	Control		Treated	Control		Treated	Control	
6 months	91 (3.9%)	71 (3.0%)	0.11	10 (0.4%)	10 (0.4%)	1.00	78 (3.3%)	84 (3.6%)	0.63
1 year	115 (4.9%)	93 (4.0%)	0.12	10 (0.4%)	13 (0.6%)	0.53	121 (5.2%)	128 (5.5%)	0.65
3 years	162 (6.9%)	118 (5.0%)	**< 0.01**	14 (0.6%)	15 (0.6%)	0.85	172 (7.3%)	215 (9.2%)	**0.02**
5 years	186 (7.9%)	124 (5.3%)	**< 0.01**	14 (0.6%)	15 (0.6%)	0.85	200 (8.5%)	240 (10.2%)	**0.05**

*Note:* Data are presented as number (percentage). Bold values indicate statistical significance set at *p* < 0.05.

Abbreviations: TgAb, thyroglobulin antibodies; TPOAb, thyroid peroxidase antibodies; TSH, thyroid stimulating hormone.

The selenium‐treated group demonstrated significantly higher mean thyroxine (T4) levels compared to the control group at all time points (*p* < 0.01), increasing from 1.6 ± 1.1 versus 1.4 ± 0.9 at 6 months to 4.1 ± 3.7 versus 3.2 ± 3.1 at 5 years (Table 3). Similarly, mean TPOAb levels were significantly higher in the treated group beginning at 1 year and remained higher at three and 5 years (*p* < 0.05), increasing from 1.4 ± 0.9 versus 1.1 ± 0.4 at 1 year to 1.8 ± 1.7 versus 1.5 ± 1.1 at 5 years. In contrast, mean TgAb levels did not differ significantly between the two groups at any time point. Mean TSH levels were also significantly higher in the selenium‐treated group at 1 year, 3 years and 5 years (*p* < 0.01), rising from 2.1 ± 1.4 versus 1.8 ± 1.3 at 1 year to 4.6 ± 4.3 versus 3.5 ± 3.3 at 5 years.

**TABLE 3:** Mean levels of thyroid markers in the selenium‐treated and control groups.

Timeline	T4			TPOAb			TgAb			TSH		
	Treated	Control	*p*	Treated	Control	*p*	Treated	Control	*p*	Treated	Control	*p*
6 months	1.6 ± 1.1	1.4 ± 0.9	**0.01**	1.2 ± 0.6	1.1 ± 0.3	0.07	1.2 ± 0.5	1.1 ± 0.3	0.22	1.5 ± 0.9	1.5 ± 1.0	0.41
1 year	2.1 ± 1.5	1.8 ± 1.2	**< 0.01**	1.4 ± 0.9	1.1 ± 0.4	**< 0.01**	1.3 ± 0.8	1.1 ± 0.4	0.12	2.1 ± 1.4	1.8 ± 1.3	**< 0.01**
3 years	3.4 ± 2.9	2.7 ± 2.4	**< 0.01**	1.7 ± 1.5	1.4 ± 1.0	**0.03**	1.5 ± 1.3	1.3 ± 0.6	0.19	3.7 ± 3.2	2.9 ± 2.5	**< 0.01**
5 years	4.1 ± 3.7	3.2 ± 3.1	**< 0.01**	1.8 ± 1.7	1.5 ± 1.1	**0.03**	1.6 ± 1.6	1.3 ± 0.7	0.11	4.6 ± 4.3	3.5 ± 3.3	**< 0.01**

*Note:* Bold values indicate statistical significance set at *p* < 0.05. Continuous data comparisons are displayed for descriptive purposes and are supported by concordant findings from categorical, distribution‐independent analyses.

Abbreviations: T4, free thyroxine; TgAb, thyroglobulin antibodies; TPOAb, thyroid peroxidase antibodies; TSH, thyroid stimulating hormone.

### 3.3 Selenium Supplementation and Risk of Autoimmune Disorders

Overall, the incidence of autoimmune disorders was significantly higher in the selenium‐treated group compared to the control group (RR 1.33, 95% CI: 1.14–1.56). Specifically, the risks of Sjögren's syndrome (RR 1.61, 95% CI: 1.03–2.52) and psoriasis (RR 2.69, 95% CI: 1.52–4.76), indicating higher risks of developing these conditions in the selenium‐treated group. For other autoimmune disorders, the differences in incidence between the selenium‐treated and control groups were not statistically significant (Table 4).

**TABLE 4:** Secondary outcomes of the present study.

Outcome	Treated	Control	*p*	RR	95% CI
Autoimmune disorders	319 (13.6%)	240 (0.1%)	**< 0.01**	1.33	(1.14, 1.56)
Rheumatoid arthritis	78 (3.3%)	57 (2.4%)	0.07	1.37	(0.98, 1.92)
SLE	31 (1.3%)	23 (1.0%)	0.27	1.35	(0.79, 2.30)
Multiple sclerosis	18 (0.8%)	10 (0.4%)	0.13	1.80	(0.83, 3.89)
Type 1 diabetes mellitus	88 (3.7%)	71 (3.0%)	0.17	1.24	(0.91, 1.69)
Celiac disease	46 (2.0%)	32 (1.4%)	0.11	1.44	(0.92, 2.25)
Pernicious anaemia	20 (0.9%)	17 (0.7%)	0.62	1.18	(0.62, 2.24)
Addison's disease	20 (0.9%)	13 (0.6%)	0.22	1.54	(0.77, 3.09)
Sjögren's syndrome	50 (2.1%)	31 (1.3%)	0.03	1.61	(1.03, 2.52)
Vitiligo	11 (0.5%)	10 (0.4%)	0.83	1.10	(0.47, 2.59)
Psoriasis	43 (1.8%)	16 (0.7%)	**< 0.01**	2.69	(1.52, 4.76)
Thyroid cancer	10 (0.4%)	13 (0.6%)	0.53	0.41	(0.15, 1.07)
Thyroidectomy	15 (42.6%)	10 (42.6%)	0.32	1.50	(0.68, 3.33)
All‐cause mortality	110 (4.7%)	56 (2.4%)	**< 0.01**	1.63	(1.18, 2.24)

*Note:* Data are presented as number (percentage). Relative risk (RR) and 95% confidence intervals (CIs) were reported. Bold values indicate statistical significance set at *p* < 0.05.

Abbreviation: SLE, systemic lupus erythematosus.

### 3.4 Risks of Thyroid Cancer and Thyroidectomy

The incidence of thyroid cancer was lower in the selenium‐treated group compared with the control group, although this difference did not reach statistical significance. Furthermore, thyroidectomy occurred more frequently in the selenium‐treated group; however, this difference was also not statistically significant (Table 4).

### 3.5 Risk of Mortality

The incidence of all‐cause mortality was higher in the selenium‐treated group compared to the control group, with the difference reaching statistical significance (RR 1.63, 95% CI: 1.18–2.24) (Table 4).

## 4 Discussion

This large‐scale retrospective cohort study investigated the effects of selenium supplementation on thyroid function, autoimmunity, and related outcomes in HT using data from a global federated health research network. The potential benefits of selenium supplementation in HT are thought to be mediated by its antioxidant and anti‐inflammatory properties. Selenium helps to reduce oxidative stress in the thyroid gland, which is believed to contribute to the development and progression of autoimmune thyroid disorders [18]. Additionally, selenium has been shown to modulate the immune response by reducing the production of pro‐inflammatory cytokines and regulating the activity of immune cells. Several studies showed that the number of Th1 and Th17 cells in the thyroid of mice was reduced following supplementation with selenium, while the number of Treg cells showed a significant increase [19, 20, 21, 22]. Indeed, other therapies ranging from wet cupping to curcumin supplementation have been shown to improve prognosis and quality of life among patients with HT through similar anti‐inflammatory mechanisms [23, 24].

Contrary to the anticipated benefits, the main findings suggest that selenium supplementation may not provide the desired beneficial effects on thyroid function and autoimmunity in patients without documented history of selenium deficiency and may even potentially be associated with adverse outcomes. In particular, selenium supplementation was associated with a higher proportion of patients with elevated TPOAb and consistently higher mean levels of free T4, TPOAb and TSH compared to the control group over the 5‐year study period. These findings challenge the notion that selenium supplementation can improve thyroid function and reduce autoimmunity in HT. The elevated TPOAb levels in the selenium‐treated group suggest a potential exacerbation of the autoimmune process, while the higher TSH levels raise concerns about the development of hypothyroidism. The increased T4 levels in the treated group may reflect a compensatory mechanism in response to the elevated TSH levels, but further investigation is needed to confirm this.

These results align with previous studies suggesting that selenium supplementation may not be as significantly beneficial as initially anticipated. Toulis et al. [25] aggregated multiple trials focusing on levothyroxine (LT4) supplementation among patients with HT and highlighted that while selenium supplementation could reduce TPOAb levels in some patients, the overall clinical benefit remained unclear. Negro et al. [26] and Wichman et al. [27] both observed reduced biomarker levels following selenium supplementation, although they were unable to identify significant improvements in clinical outcomes.

On the other hand, there exist multiple meta‐analyses aggregating data from a variety of randomised clinical trials that support selenium supplementation in affected patients. Zuo et al. and Zhang et al. showed significant reductions in thyroid biomarkers associated with selenium supplementation, with the latter finding correlations between this and improvements in well‐being and mood [28, 29]. Similarly, Huwiler et al. [13] noted reductions in the same biomarkers, further observing that the majority of reductions occurred in patients, regardless of them taking or not taking thyroid hormone replacement therapy. Although van Zuuren et al. [30] found significant decreases as well, they lacked sufficient evidence to confirm the clinical efficacy of selenium supplementation in affected patients.

Such heterogeneity within these studies, as well as between them and the present study, may reflect geographic differences in dietary selenium intake and baseline selenium levels. Indeed, dietary selenium is generally based on the levels of selenium within the soil content of a region [31]. This may explain why both dietary intake and baseline serum levels of the nutrient are suboptimal in China and European nations, and widely variable but suboptimal nonetheless in Middle Eastern nations, especially when compared to the United States [32, 33]. Although the current study excluded patients with selenium deficiency, it used de‐identified data and was unable to stratify patients based on geographical status. As such, there is a great need to further investigate this across diverse populations to clarify the impact of supplementation. In particular, while TPOAb reduction suggests a potential immunomodulatory effect as described in the aforementioned studies, the absence and variations of observed tangible clinical benefits imply that further research is warranted to evaluate meaningful clinical endpoints and determine if these biomarker changes translate into clinically relevant benefits.

The study did not find a significant impact of selenium supplementation on the incidence of thyroid cancer. While these findings are reassuring, the relatively low incidence of these outcomes in the study population may have limited power to detect significant differences between the groups. These findings differ from previously mentioned studies that reported beneficial effects of selenium supplementation [25, 26]. Indeed, differences in study design, population characteristics, selenium dosage, and duration of supplementation could explain these discrepancies.

Notably, the present study found a significantly higher incidence of autoimmune disorders, particularly Sjögren's syndrome and psoriasis, in the selenium‐treated group compared to the control group. This finding raises concerns that selenium supplementation may trigger or exacerbate other autoimmune conditions in affected patients with HT. It is plausible that selenium's immunomodulatory effects may have unintended consequences in genetically susceptible individuals, leading to the development of additional autoimmune disorders [34].

Furthermore, the significantly higher incidence of all‐cause mortality in the selenium‐treated group at 5 years and overall raises concerns about the long‐term safety of selenium supplementation in this patient population. While the exact reasons for this increased mortality risk are not clear from the current study, the exacerbation of autoimmunity and the development of additional autoimmune disorders may potentially contribute to the observed mortality difference. On the other hand, recent evidence has shown that selenium supplementation may be beneficial regarding other indications, including healing bedsore ulcers, modulation of myocardial inflammation following cardiac surgery, and chemoprevention of bladder cancer [35, 36, 37]. Considering the conflicting results throughout the literature as well as the variety of applications, further research is warranted to explore selenium's role and mechanisms of action fully.

Certain limitations must be acknowledged. As a retrospective study, the present study cannot establish a causal relationship between selenium supplementation and the observed outcomes. Additionally, there is always the potential for misclassification when utilizing an administrative database; as such, there is no absolute guarantee that all patients with selenium deficiency were excluded, especially as serum selenium levels were unavailable. However, it is likely that the study population was already selenium‐sufficient, which may explain the lack of observed therapeutic effect. The study population was also derived from multiple countries which may encompass different dietary patterns, baseline nutritional status, and variable selenium intake, all which may confound the observed associations. Furthermore, although selenium use was defined as at least 6 months of supplementation, it remains uncertain whether this duration is sufficient to meaningfully influence long‐term clinical outcomes. Finally, the study did not have information on the dose, duration, or adherence to selenium supplementation, which could have influenced the results.

Limitations specifically regarding the statistical methodology must also be appreciated. The federated nature of TriNetX provides aggregated data which precludes the independent verification of raw data distribution through formal tests such as Shapiro–Wilk or Levene's test for homogeneity of variance. As such, the built‐in student's *t*‐test was utilised as the primary tool for continuous variables without the option for Welch‐correction. However, to ensure the robustness of our findings, we cross‐validated these findings with categorical analyses using Chi‐square tests. The high degree of concordance between these parametric and non‐parametric methods suggests that the distributional characteristics of the raw data did not significantly impact the clinical interpretations. Nevertheless, the present study's strengths include a large sample size, the use of propensity score matching to balance potential confounders between the treated and control groups, and the long‐term follow‐up period. Of course, further research must address the above limitations to meaningfully guide clinical decision‐making.

## 5 Conclusion

The present study found that selenium supplementation in patients with HT was associated with higher levels of TPOAb, T4 and TSH, an increased incidence of autoimmune disorders, and a higher risk of all‐cause mortality compared to the control group. These findings challenge the supplementation of this nutrient as a therapeutic option for patients with HT, especially if they were not deficient to begin with. Healthcare providers should exercise caution when considering selenium supplementation for patients with HT and closely monitor patients for signs of thyroid dysfunction, autoimmunity and other potential complications.

## Author Contributions

**Claire Ardis:** investigation, writing – original draft. **Rami M. Elshazli:** formal analysis, writing – original draft. **Eman A. Toraih:** conceptualization, data curation, investigation, writing – review and editing. **Ekramy M. Elmorsy:** data curation, formal analysis, writing – original draft. **Mohammad H. Hussein:** validation, writing – review and editing. **Hinali Patel:** data curation, formal analysis, writing – original draft. **Manal S. Fawzy:** conceptualization, project administration, resources, supervision, validation, writing – review and editing. **Sarah Yaghi:** conceptualization, data curation, formal analysis, methodology, software, writing – original draft. **Jessan A. Jishu:** validation, software, writing – review and editing. **Lori Tran:** investigation, writing – original draft. **Hani Aiash:** project administration, resources, writing – review and editing, supervision. **Ahmed Abdelmaksoud:** data curation, methodology, writing – original draft.

## Funding

The authors have nothing to report.

## Ethics Statement

The study involved a retrospective analysis using the database with de‐identified study subjects, thereby exempting it from needing institutional review board (IRB) approval.

## Conflicts of Interest

The authors declare no conflicts of interest.

## Supporting information


**Table S1:** Inclusion and exclusion criteria for patient selection.
**Table S2:** Definitions of primary and secondary outcomes in the present study.

## Data Availability

The data that support the findings of this study are available from the corresponding author upon reasonable request.
