# Supplementary material for: Clinical Outcomes of Selenium Supplementation in Hashimoto's Thyroiditis Without Selenium Deficiency: A Large‐Scale Retrospective Cohort Study
Source: Endocrinol Diabetes Metab. 2026 May 18;9(3):e70239. doi: 10.1002/edm2.70239 (PMC13184170; doi:10.1002/edm2.70239)
Supplement: Supplementary file 1 — Table S1: Inclusion and exclusion criteria for patient selection. Table S2: Definitions of primary and secondary outcomes in the present study. [file EDM2-9-e70239-s001.docx]

**Supplementary Table S1.** Inclusion and exclusion criteria for patient selection.

| **Category** | **Code** | **Content** |
| --- | --- | --- |
| Inclusion criteria | |  |
| *Autoimmune Thyroiditis* | |  |
| Diagnosis | ICD-10-CM: E06.3 | Hashimoto Thyroiditis |
| *Selenium Supplementation* | |  |
| Medication | ATC: A12CE | Selenium |
| Medication | RXNORM: 9641 | Selenium |
| *Exclusion criteria* |  |  |
| Demographics | Age | Age < 18 years |
| Diagnosis | ICD-10-CM: E59 | Dietary selenium deficiency |
| Diagnosis | ICD-10-CM: C00-D49 | Neoplasms |
| Procedure | SNOMED: 17198800 | Excision of lesion of the thyroid |
| Procedure | ICD-10-PCS: 0GBJ | Endocrine System / Excision / Thyroid Gland Isthmus |
| Procedure | ICD-10-PCS: 0GBG | Endocrine System / Excision / Thyroid Gland, Left |
| Procedure | ICD-10-PCS: 0GBH | Endocrine System / Excision / Thyroid Gland, Right |

**Supplementary Table S2.** Definitions of primary and secondary outcomes in the present study.

| **Category** | **Code** | **Content** |
| --- | --- | --- |
| **Primary outcomes** | |  |
| *Thyroid peroxidase levels* | |  |
| Laboratory | LNC: 8099-4 | Thyroperoxidase Ab [Units/volume] in Serum or Plasma (at least 35.00 [IU]/mL (most recent occurrence)) |
| Laboratory | LNC: 56477-3 | Thyroperoxidase Ab [Units/volume] in Serum or Plasma by Immunoassay (at least 35.00 [IU]/mL (most recent occurrence)) |
| Laboratory | LNC: 32786-6 | Thyroperoxidase Ab [Titer] in Serum or Plasma (at least 35.00 1:N [titer] (most recent occurrence)) |
| Laboratory | LNC: 18332-7 | Thyroperoxidase IgG Ab [Units/volume] in Serum or Plasma (at least 35.00 [arb'U]/mL (most recent occurrence)) |
| Laboratory | LNC: 63361-0 | Thyroperoxidase Ab [Units/volume] in Body fluid by Immunoassay (at least 35.00 [IU]/mL (most recent occurrence)) |
| Laboratory | TNX: LG359-6 | Thyroperoxidase Ab [Units/volume] in Serum, Plasma, or Blood (at least 35.00 [IU]/mL (most recent occurrence)) |
| *Thyroglobulin level* | |  |
| Laboratory | LNC: 8098-6 | Thyroglobulin Ab [Units/volume] in Serum or Plasma |
| *Thyroxine Level* | |  |
| Laboratory | LNC: 3024-7 | Thyroxine (T4) free [Mass/volume] in Serum or Plasma |
| *TSH level* |  |  |
| Laboratory | LNC: 3016-3 | Thyrotropin [Units/volume] in Serum or Plasma (most recent occurrence) |
| **Secondary outcomes** | |  |
| *Autoimmune disorders* | |  |
| Diagnosis | ICD-10-CM: M05 | Rheumatoid arthritis with rheumatoid factor |
| Diagnosis | ICD-10-CM: M06 | Other rheumatoid arthritis |
| Diagnosis | ICD-10-CM: M32 | Systemic lupus erythematosus (SLE) |
| Diagnosis | ICD-10-CM: G35 | Multiple sclerosis |
| Diagnosis | ICD-10-CM: E10 | Type 1 diabetes mellitus |
| Diagnosis | ICD-10-CM: K90.0 | Celiac disease |
| Diagnosis | ICD-10-CM: D51.0 | Vitamin B12 deficiency anemia due to intrinsic factor deficiency |
| Diagnosis | ICD-10-CM: M35.0 | Sjögren syndrome |
| Diagnosis | ICD-10-CM: L80 | Vitiligo |
| Diagnosis | ICD-10-CM: L40 | Psoriasis |
| Diagnosis | ICD-10-CM: E27.1 | Primary adrenocortical insufficiency |
| *Thyroid cancer* | |  |
| Diagnosis | ICD-10-CM: C73 | Malignant neoplasm of the thyroid gland |
| Diagnosis | ICD-10-CM: Z85.850 | Personal history of malignant neoplasm of the thyroid gland |
| *Thyroidectomy* | |  |
| Procedure | SNOMED: 13619001 | Thyroidectomy |
| Procedure | CPT: 60240 | Thyroidectomy, total or complete |
| Procedure | CPT:1009039 | Thyroidectomy, total or subtotal for malignancy |
| Procedure | CPT: 60252 | Thyroidectomy, total or subtotal for malignancy, with limited neck dissection |
| Procedure | CPT:1009043 | Thyroidectomy, including substernal thyroid |
| Procedure | CPT: 60260 | Thyroidectomy removal of all remaining thyroid tissue following previous removal of a portion of the thyroid |
| Procedure | CPT: 60271 | Thyroidectomy, including substernal thyroid; cervical approach |
| Procedure | CPT: 60254 | Thyroidectomy, total or subtotal for malignancy, with radical neck dissection |
| Procedure | CPT: 60270 | Thyroidectomy, including substernal thyroid; sternal split or transthoracic approach |
| Procedure | SNOMED: 359882009 | Thyroidectomy with laryngectomy |
| Procedure | SNOMED: 24443003 | Total thyroidectomy |
| Procedure | SNOMED: 30956003 | Subtotal thyroidectomy |
| Procedure | SNOMED: 52826006 | Substernal thyroidectomy |
| Procedure | SNOMED: 24711004 | Partial substernal thyroidectomy |
| Procedure | SNOMED: 237486002 | Total thyroidectomy with cervical lymph node dissection |
| Procedure | ICD-9-CM: 06.5 | Substernal thyroidectomy |
| Procedure | SNOMED: 712978001 | Revision thyroidectomy |
| Procedure | SNOMED: 719753008 | Completion thyroidectomy |
| Procedure | SNOMED: 767574004 | Operation on the parathyroid gland during thyroidectomy |
| Procedure | ICD-10-PCS: 0GBG | Endocrine System / Excision / Thyroid Gland Lobe, Left |
| Procedure | ICD-10-PCS: 0GBH | Endocrine System / Excision / Thyroid Gland Lobe, Right |
| *All-cause mortality* | |  |
| Demographics | Deceased | Deceased |
| Diagnosis | ICD-10-CM: R99 | Ill-defined and unknown cause of mortality |
